# Temporomandibular disorder is associated with a serotonin transporter gene polymorphism in the Japanese population

**DOI:** 10.1186/1751-0759-1-3

**Published:** 2007-01-10

**Authors:** Kiyomi Ojima, Nagaoki Watanabe, Naoko Narita, Masaaki Narita

**Affiliations:** 1 Graduate School Human Sciences, University of Tsukuba, Ibaraki, Japan; 2 Department of Oral and Maxillofacial Surgery, Kawaguchi Municipal Medical Center, Saitama, Japan

## Abstract

**Aims:**

Recent genetic studies have linked serotonin-related genetic polymorphisms with diverse disorders characterized by functional somatic symptoms, including chronic fatigue syndrome, irritable bowel syndrome, and premenstrual dysphoric disorder.

**Methods:**

We investigated three serotonin-related genetic polymorphisms by screening genomic DNA of 36 temporomandibular disorder (TMD) patients.

**Results:**

A significant increase of longer alleles (l and xl) was found in the TMD patients compared to the controls both by the genotype-wise and the allele-wise analyses (both p < 0.01 by χ^2 ^test and Fisher's exact test).

**Conclusion:**

Genetic factors that involve the serotonergic system may play a role in the pathogenesis of TMD.

## Findings

Temporomandibular disorder (TMD) is a heterogeneous group of disorders affecting the temporomandibular joints, the masticatory muscles, or both. TMD generally develops orofacial pain as an initial symptom, and approximately 10 % of all orofacial pain patients who complain of localized myalgia or tenderness in the masticatory muscles for more than one month are generally diagnosed as TMD [1,2]. TMD is often comorbid with other psychosomatic symptoms such as sleep disorder, headache, fatigue, and depression, which are characterized as functional somatic syndromes (FSS) [3,4]. The concept of FSS is a multidimensional classification based on clinical features, and it incorporates a variety of syndromes such as chronic fatigue syndrome (CFS), irritable bowel syndrome (IBS), premenstrual dysphoric disorder (PMDD), TMD, and non-cardiac chest pain. Although the physiological function of neurotransmitter serotonin (5-hydroxytryptamine, 5-HT) accounts for most of these common symptoms of FSS, the concept itself has been controversial to date because of the lack of a biological etiology to explain the mechanism of FSS [5]. The 5-HT neuronal system regulates diverse physiologic functions including sleep, respiration, appetite, pain, motor function, cognition, sexual activity, as well as emotions such as mood and anxiety. Polymorphic study within 5-HT related genes are reported to be associated with numerous disorders and illness by our group and others, including CFS [6], IBS [7,8], and PMDD [9,10], suggesting a relevance of 5-HT neuronal dysfunction to the disease categorized as FSS. Therefore, we screened three 5-HT related genomic DNA polymorphisms in patients with TMD, another disorder categorized as FSS.

Thirty-six patients (mean age; 45.3+/-16.7 years old, 5 males and 31 females) at the Department of Oral and Maxillofacial Surgery of Kawaguchi Municipal Medical Center were enrolled in the study. Patients were diagnosed as TMD by chronic and localized myalgia and tenderness to palpation that persisted for more than one month, basically according to the literatures describing research diagnostic criteria for TMD. (Anastassaki and Magnusson 2004). For controls, 119 healthy volunteers (mean age 26.4+/- 8.9 years old, 49 men and 70 women) were recruited to the study. Written informed consent was obtained prior to the study, and the study was approved by the ethics committees of Kawaguchi Municipal Medical Center and the University of Tsukuba.

Genomic DNA was extracted from the whole blood of every participant using a Genomic DNA isolation reagent (E.Z.N.A Blood DNA Kit?, Omega Bio-tek, GA, USA). The extracted DNA was amplified by polymerase chain reaction (PCR) to detect serotonin-related polymorphisms. To avoid bias in the observation, all DNA samples were tested without sample information until all data was obtained.

For serotonin transporter protein promoter polymorphism (5-HTTLPR) analysis, extracted DNA was amplified by polymerase chain reaction (PCR), basically according to the method of Lesch et al. [11]. Oligonucleotide primers flanking the 5-HTTLPR and corresponding to the nucleotide positions-1416 to -1397 (5'-GGCGTTGCCGCTCTGAATGC) and -910 to 88 (5'-GAGGGACTGAGCTGGACAACCAC) of the 5-HTT gene 5'-flanking regulatory region were used to generate 484(s)-, 528(l)-, 572 or 616(xl) – bp fragments.

For 5HTTintron2 variable-number-tandem-repeat (VNTR), oligonucleotide primers set within intron 2 to detect the VNTR region, 5'-GTCAGTATCACAGGCTGCGAG-3' and 5'-TGTTCCTAGTCTTACGCCAGTG-3' were used as described by Collier et al [12]. The amplified products were either 250 or 284 bp fragments, corresponding to the 9 and 11 repeat-containing alleles, respectively.

For -1438 G/A serotonin 2A receptor (5HT 2AR), oligonucleotide primers set within the 5HT 2A receptor gene 5'-flanking promoter region, 5'-AAGCTGCAAGGTAGCAACAGC-3' and 5'-AACCAACTTATTTCCTACCAC-3' were used as previously described by Collier et al [13], followed by digesting the amplified 468 bp products with Msp I (Takara bio inc, Japan), which cuts the -1438/G allele into two fragments of 244 and 224 bp. The end products were either 468 bp, corresponding to -1438/A allele, or 244 and 224 bp corresponding to -1438/G allele.

All PCR amplification was carried out in a final volume of 20 μl consisting of 50 ng of genomic DNA, 2.5 mM deoxyribonucleotides, 20 pmol of forward and reverse primers, 10 mM tris/HCl (pH 8.3), 50 mM KCl, 1.5 mM MgCl_2_, and 1 U of Taq DNA polymerase (Takara bio inc, Japan). Annealing was carried out at 61°C for 30 s, extension at 72°C for 1 min, and denaturation at 95°C for 30 s for 35 cycles. PCR products were visualized by 2 % agarose gel electrophoresis followed by ethidium bromide staining.

Statistical analysis was done for comparisons of the TMD and the control groups. χ^2 ^test (2-tailed) was used for between group comparisons of the genotype distribution and allele frequency of gene polymorphism. Fisher's exact test was also used to examine the *p *value. *P *values less than 0.05 were considered to be statistically significant.

Table 1 shows a summary of the genotype distribution analysis of 5HTTLPR. Genotypes including longer alleles (l and xl) were statistically increased in the TMD group compared to control group (*p *= 0.0079 by Pearson's χ^2 ^test and *p *= 0.0044 by Fisher's exact test) (upper panel). When 5-HTTLPR was analyzed allele-wise, the longer alleles were found in 14.3% (13.9% l allele and 0.4% xl allele, respectively) of the control subjects (Table 1, lower panel). Statistical analysis revealed a significant difference between the patient and control groups (*p *= 0.0027 by Pearson's χ^2 ^test and *p *= 0.0019 by Fisher's exact test). 5HTTintron2VNTR or -1438G/A5HT2AR polymorphic analysis was also performed, however, no significant difference was found between the patients and controls by either genotype distribution analysis or allele frequency analysis (data not shown).

**Table 1:** Genotype distribution data (upper panel) and allele frequency data (lower panel) for 5HTTLPR from TMD patients and controls.

**genotype**	**TMD**	**control**
5HTTLPR	n = 36	n = 119
s/s	17 (47.2%)	87 (73.1%)
s/l	14 (38.9%)	29 (24.4%)
l/l	4 (11.1%)	2 (1.7%)
s/xl	1 (2.8%)	1 (0.8%)
p value		
Pearson's χ^2 ^test	0.0079	
Fisher's exact test	0.0044	

**allele**	**TMD**	**control**

5HTTLPR	n = 72	n = 238
s	49 (68.1%)	204 (85.7%)
l	22 (30.5%)	33 (13.9%)
xl	1 (1.4%)	1 (0.4%)
p value		
Pearson's χ^2 ^test	0.0027	
Fisher's exact test	0.0019	

The l allele of 5-HTTLPR, which was the most frequently observed in the TMD patients in the present study, is supposed to retain higher transcriptional activity than the S allele [11]. This may result in a lower concentration of 5-HT in the extracellular space, namely, active 5-HT. To date, a number of diseases have been proved to be associated with 5-HTTLPR. For instance, the longer alleles (l and xl) were demonstrated to be significantly increased in the victims of sudden infant death syndrome (SIDS) [14-16]. This fact may explain the pathophysiology of SIDS, which has long been suspected to be a defect in the arousal or sleep mechanisms in the brainstem regulated by the serotonergic neuronal system. Anxiety trait [11], mood disorder [17, 18, 19, 20], autism [19, 21], and eating disorders have likewise been investigated in an attempt to show an association with 5-HTTLPR [22, 23], although much remains to be clarified and some of the results are controversial.

The definition of FSS allows for a substantial overlap in the symptoms of syndromes such as CFS, IBS, and fibromialgia (FM). Therefore, patients may have more than one diagnosis, depending on the expertise of the physician they visit. It is more persuasive that FSS may be caused by a dysfunction of central nervous system, especially of the 5-HT neuronal system, rather than by the abnormalities in specific organ systems. This may explain the multiple symptoms of FSS and the frequent co-morbidities of emotional disorders such as anxiety disorder and major depression.

Some replicated results of challenge tests with CFS patients have been reported, including enhanced prolactin response to a selective 5-HT agonist, D-fenfluramine. Since the interaction between the hypothalamo-pituitary-adrenal axis and the 5-HT system is probably mediated partially by the 5-HT receptors in the hippocampus, this result indicates a hypofunction of the 5-HT system and/or a hypersensitivity of the 5-HT receptors in the brain of CFS patients [24, 25, 26]. Genetic association among FSS diseases have also been reported. For example, longer alleles (l and x/l) are frequent in CFS [6], s/s genotype was increased in diarrhea predominant IBS [7], whereas the l/l genotype was increased in constipation type IBS [7,8]. PMDD is also reported to be associated with 5HTTLPR [9]. Although the increased allele is different depending on the disease, these reports indicate that serotonergic dysfunction might be involved in the pathogenesis of these diseases because the polymorphism is functional.

Our results clearly show a significant association between the longer alleles (l and xl) of 5HTTLPR and the TMD group. This facts suggest to us that 1; 5HTTLPR could be further exploited as a diagnostic tool for TMD out from other orofacial pain diseases. 2; the pathogenesis of TMD is thought to be significantly associated with serotonergic neuronal dysfunction, which is in common with other disorders as FSS. To our knowledge, this is the first reported genetic linkage to TMD, and it emphasizes the '5-HT system dysfunction hypothesis' when considering the etiology of the disease. Considering TMD to include overlapping FSS syndromes such as CFS and IBS, we can postulate that individuals carrying the longer alleles are more susceptible to FSS than individuals with the short allele, because of the relative hypofunction of the 5-HT system due to a lower 5-HT concentration in the extracellular space, compared to the ones with the short allele. However, we have to be very careful in our evaluation of these findings because of the extremely small sample size, even though the *p *value is small. Further independent studies will be necessary to confirm the present data.

## Authors' contributions

KO carried out the molecular genetic studies and performed the statistical analysis. NW performed clinical diagnosis. NN participated in the design of the study and drafted the manuscript. MN conceived the study and participated in its design and coordination and helped to draft the manuscript. All authors read and approved the final manuscript.
